# Dilatation of the Cervical Canal for Spasmodic Dysmenorrhœa and Sterility

**Published:** 1882-05

**Authors:** 


### Dilatation of the Cervical Canal for Spasmodic Dysmenorrhœa and Sterility.

Dr. Godson prefaced his remarks on dilatation of the cervical canal for spasmodic dysmenorrhœa and sterility, at a recent meeting of the Obstetrical Society of London, by sketching the history of the method, introduced more than fifty years ago by Dr. Mackintosh, of Edinburgh, and lately advocated by Dr. Matthews Duncan. Out of ten patients suffering from dysmenorrhœa with sterility, treated by the author, five had become pregnant. The dysmenorrhœa was characterized by severe colicky pains in the hypogastric and sacral regions, either before the flow or coincident with it. He preferred to drop the term "obstructive," as he knew of no evidence that there was want of patency of the cervical canal, and Duncan had passed a probe into the uterus at the height of the pain without meeting with obstruction. He believed that spasm of the uterine muscular tissue was enough of itself to give rise to the severe pain, without any obstruction. After giving brief notes of five cases, the author gave the following conclusions: 1. That the method was simpler and safer than any other proposed. 2. That the dilatation might be done safely at the physician's house. 3. That a very small amount of dilatation was necessary. 4. That it should be done a week or ten days after a menstrual period. 5. That it should be done, not on successive days, as hitherto recommended, but all at once; that the first bougie should be a small one, and that there should not be enough difference in size between the successive bougies to split the mucous membrane. 6. That pregnancy appeared to take place on account of the dilatation having cured the conditions on which the dysmenorrhœa depended. In none of the author's cases was there either stenosis or constriction of the canal by acute flexion. In one instance the relief of the dysmenorrhœa proved only temporary, and a cure was afterward effected with an intra-uterine stem. One patient was lost sight of; the three others (of the five whose sterility was not cured) were relieved of dysmenorrhœa. . . . Dr. Graily Hewitt regarded dilatation as unnecessary in cases in which the uterus was unduly soft and pliable, but in long-standing cases it was of great assistance. He used a two-bladed dilatator, acting like a glove-stretcher. . . . Dr Heywood Smith thought it best to have the uterine portion of the instruments straight, not curved as in the dilators shown. . . . Dr. Routh did not see what advantage the method had over that by dilatation with laminaria tents, followed by the employment of an intra-uterine stem. An analogous plan had formerly been used at the Samaritan Hospital, but had been abandoned, proving not to be so free from danger as had been stated. The effect was transitory, unless pregnancy occurred very soon, and the pain induced was sometimes very great. In cases of flexion it was often difficult to pass even a bent sound, and the use of a straight dilator in such cases would be liable to set up inflammation.—*N. Y. Med. Journ. and Obst. Rev.*

